# Perioperative Changes in Renal Resistive Index as a Predictor of Acute Kidney Injury After Cardiac Surgery: A Prospective Cohort Study

**DOI:** 10.3390/jcm14176315

**Published:** 2025-09-07

**Authors:** Marie Sabia, Christian Isetta, Rishika Banydeen, Nicolas Durand, Hossein Mehdaoui, Marc Licker

**Affiliations:** 1 Department of General Critical Care, University Hospital of Martinique, F-97200 Fort-de-France, France; 2 Department of Cardiovascular & Thoracic Anesthesia and Critical Care, University Hospital of Martinique, F-97200 Fort-de-France, France; isetta.christian0774@orange.fr (C.I.); marc.licker@unige.ch (M.L.); 3 Department of Clinical Research, University Hospital of Martinique, F-97200 Fort-de-France, France; rishika.banydeen@chu-martinique.fr; 4 Department of Anesthesia & Intensive Care, Cardio-Thoracic Center, F-98004 Monaco, France; 5 Faculty of Medicine, University of Geneva, CH-1206 Geneva, Switzerland

**Keywords:** renal resistive index, acute kidney injury, cardiac surgery, Doppler ultrasound

## Abstract

**Background:** Cardiac surgery-associated acute kidney injury (CSA-AKI) is common and various tools are proposed to identify patients at risk of AKI. The determination of the Doppler-derived renal resistance index (RRI) is useful for detecting the occurrence of tubular necrosis or allograft rejection. This study questions the value of RRI in identifying CSA-AKI, defined according to the renal risk, injury, failure, loss of kidney function, and end-stage kidney disease (RIFLE) classification. **Methods:** We conducted a prospective, unblinded, observational study in patients undergoing open heart surgery. Clinical and surgical data were collected from the electronic medical files and the Cleveland score was calculated for each patient. Before the surgery and upon admission to the intensive care unit (ICU), blood flow in the renal cortical or arcuate arteries was measured and the RRI was computed. The capability of preoperative serum creatinine, the Cleveland score, and the preoperative and postoperative change in RRI were investigated with the area under the receiver operating characteristic curve (ROC-AUC) to predict the AKI. **Results:** Within the first five postoperative days, 31.4% developed CSA-AKI. All patients with stage 1 AKI recovered normal creatinine levels before ICU discharge while those with stage 2 or 3 (AKI 2/3) exhibited persistent changes. To discriminate AKI 2/3, the ROC-AUC was less than 0.7 for the preoperative serum creatinine and RRI, 0.879 for the Cleveland score, and 0.710 for the postoperative RRI. The change between the preoperative and postoperative RRI (dRRI) provided a ROC-AUC of 0.825 (sensitivity 72.7% and specificity 96.6%) with an optimal cut-off point at 9.4%. **Conclusions:** Noninvasive determination of RRI is helpful for detecting PO-AKI and provides additional information to clinical markers.

## 1. Introduction

Cardiac surgery-associated acute kidney injury (CSA-AKI) occurs in 5 to 50% of patients, resulting in prolonged hospital length of stay, high medical costs, and poor outcomes [1]. Changes in plasma creatinine levels and urine output are used to define AKI with its most severe form requiring renal replacement therapy, in 5–13% of critically ill patients [2]. A variety of novel serum and urinary biomarkers, such as neutrophil gelatinase-associated lipocalin (NGAL), kidney injury molecule-1 (KIM-1), liver-type fatty acid-binding protein (L-FABP)), interleukin-18 (IL-18)), and tissue inhibitor of metalloproteinases-2  associated with insulin-like growth factor-binding protein-7 (TIMP-2 × IGFBP-7) [3] have been tested to predict and/or detect subclinical forms of AKI [4]. Since CSA-AKI increases morbidity and mortality, research efforts have been focused on the prevention, personalized risk stratification of AKI, and development of reno-protective interventions [5].

Several predisposing factors of CSA-AKI have been identified, such as cardiovascular and pulmonary diseases, nephrotoxic agents (i.e., nonsteroidal anti-inflammatory drugs, inhibitors of the renin-angiotensin system, X-ray contrast agents), and prolonged cardiopulmonary bypass (CPB) and aortic clamp time [6].

During cardiac surgery, multiple renal stressors are acting as a result of the intense neuro-endocrine and inflammatory response, ischemia–reperfusion injuries, non-pulsatile blood flow, and exposure to artificial materials and homologous blood products [6]. Other than low cardiac output syndrome (LCOS) and episodes of hypoxemia, intrarenal vasoconstriction consequent to the release of endogenous mediators (i.e., thromboxane, endothelin, vasopressin, angiotensin II) and/or the administration of vasopressors further contribute to the impairment of the macro- and microvascular flow leading to ischemic glomerular and tubular injuries [6].

In critically ill patients, renal vasoconstriction is an early manifestation of AKI [7]. Using the Doppler ultrasound, the renal resistive index (RRI) is computed as the ratio of the difference between the maximum and minimum flow velocity. This readily available parameter reflects the alteration in the blood flow profile within the intrarenal arcuate and interlobar arteries. Elevated RRI is frequently reported in elderly patients, those with atherosclerosis, arterial hypertension, diabetes mellitus, chronic kidney disease, and rejection of renal allograft [8,9].

In the current study, we measured the renal arterial blood flow before surgery and upon admission to the intensive care unit (ICU) and we questioned whether the determination of RRI could predict the development of AKI within the first five days after cardiac surgery.

## 2. Methods

### 2.1. Study Design and Participants

This was a prospective, observational, clinical study that included adult patients (≥18 years) undergoing on-pump cardiac surgery at the University Hospital of Fort-de-France, in Martinique. The study was approved by the Committee on Health Research Ethics (Comité de protection des personnes, University of Bordeaux) and was conducted in accordance with the STROBE guidelines outlined in the declaration of Helsinki. All participants gave informed signed consent. Preoperative end-stage renal insufficiency (dialysis or hemofiltration) and poor abdominal echogenicity or no visualization of the renal vessels were considered exclusion criteria. Patients requiring extracorporeal life support for weaning from CPB were secondarily excluded due to the non-pulsatile flow pattern.

### 2.2. Clinical, Hemodynamic, and Laboratory Variables

Demographic, clinical, and surgical data were collected from the hospital electronic medical files. To evaluate the risk of postoperative AKI, the Cleveland score was computed based on the preoperative information (gender, previous cardiac surgery, chronic obstructive lung disease, diabetes mellitus, heart failure, left ventricular ejection fraction <35%, use of intraaortic balloon pump [IABP], and serum creatinine level) [10]. To assess the overall risk of perioperative mortality, the European System Operative Score Risk Evaluation score (EuroSCORE II) was calculated. The mean arterial pressure (MAP), heart rate (HR), central venous pressure, central venous oxygen saturation (ScvO_2_), and hematocrit were measured after anesthesia induction and at the end of surgery. At the time of the study, our hospital laboratory used the Cockroft–Gault equation to estimate glomerular filtration rate (eGFR-CG) based on age, gender and weight. A posteriori and in accordance with more recent guidelines, we also used the Modification of Diet in Renal Disease (MDRD) equation to compute eGFR [11].

### 2.3. Perioperative Procedure

General anesthesia with intravenous administration of propofol, sufentanil, and rocuronium was targeted to maintain a bispectral index between 40 and 60 and MAP in the range of +/−20% of preoperative values (and above 60 mmHg). For antibiotic prophylaxis, cefazoline was administered upon induction of anesthesia. Surgical procedures were conducted under cold blood cardioplegia and closed-circuit cardiopulmonary bypass (CPB). After aortic unclamping and recovery of satisfactory spontaneous heart rate rhythm or pacing-stimulated heart rate, weaning from CPB was guided by transoesophageal echocardiography under optimal volume loading conditions [12]. Inotropes (dobutamine, epinephrine) and noradrenaline infusion were initiated in the presence of new/worsening ventricular dysfunction and/or low MAP (<70 mmHg) that was not responsive to fluid loading and optimization of heart rhythm. Conversely, the decision to withdraw inotropic support was taken if the hemodynamic condition improved steadily. Protective lung ventilation was applied and patients were extubated within the first 6 h after admission to the ICU. Transfusion of red blood cells was given to maintain hemoglobin levels above 70 g/L.

### 2.4. Study Timeline

On the last weekday preceding surgery, a baseline ultrasound examination was performed, blood samples were taken, and demographic patient characteristics were obtained. The ultrasound examination was repeated within 1 h upon arrival to the ICU under continuous sedation and mechanical ventilation. Serum creatinine was measured daily after surgery until the fifth postoperative day or until discharge of the patients with AKI.

### 2.5. Ultrasound Examination and Measurements

**Figure 1:**
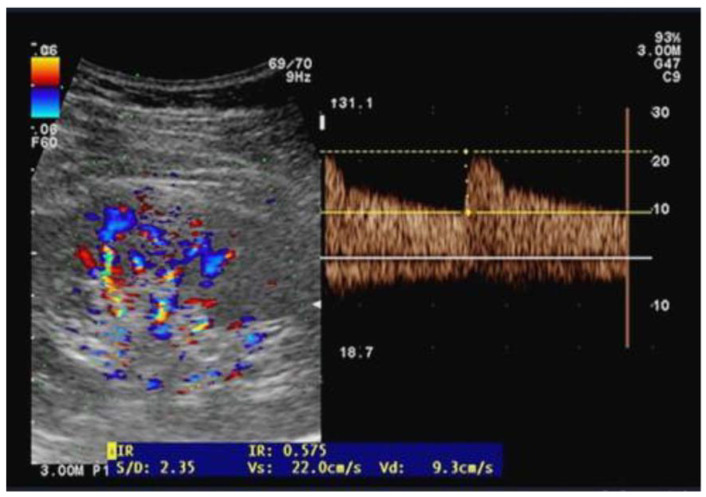
Illustration of an arterial flow measurement using the Doppler pulse velocity to derive the renal resistive index.

Ultrasound examinations were performed by two independent well-trained sonographers who were not involved in patient care while the anesthesia and ICU physicians were blinded to the results of the renal Doppler measurements. The same operator performed pre- and postoperative examinations on the same patients placed in the supine position and using a CX-50 ultrasound machine with a curvilinear 5 MHz pulsed-wave Doppler flow probe (Philips Healthcare, Eindhoven, Netherland). On each kidney, recordings with the best imaging quality were selected in the longitudinal axis. Pulsed wave Doppler curves in the interlobar or arcuate arteries were recorded with a sample volume of 2–4 mm, three measurements were performed on each kidney and then averaged (Figure 1). The perioperative change in RRI (dRRI) was computed as the ratio of the difference between preoperative and postoperative RRI to postoperative RRI (dRRI = [preoperative RRI—postoperative RRI]/preoperative RRI).

### 2.6. Study Endpoints

CSA-AKI was defined by the change in the postoperative plasma creatinine relative to preoperative measurements, according to the Risk, Injury, Failure, Loss of kidney function, and End-stage kidney disease (RIFLE) classification (Table 1) [13]. Plasma creatinine taken on the last weekday before surgery served as the baseline reference. Based on the maximum plasma creatinine within the first five postoperative days, CSA-AKI was further categorized into AKI severity levels: no injury (AKI-0), mild or stage 1 AKI (Risk, stage AKI-1), moderate or stage 2 AKI (AKI-2), and severe or stage 3 (AKI-3).

The use of vasoactive medication, durations of cardiopulmonary bypass, and aortic cross-clamping as well as ICU and hospital stay were retrieved from the patient’s medical file. An LCOS was defined by impaired ventricular contractility despite appropriate volume loading that requires the administration of at least 1 inotrope (dobutamine, epinephrine) over at least 6 h [14].

### 2.7. Statistical Analysis

Given the exploratory nature of the study and scant data in the literature, it was not possible to formulate a hypothesis and perform a power analysis for sample size calculation. Due to the small number of patients, data from AKI-0 and AKI-1 were merged as well as those from AKI-2 and AKI-3, to distinguish between mild or absent AKI and moderate to severe AKI.

Patient and clinical characteristics were compared using Student *t*-tests or Mann–Whitney tests for continuous variables when appropriate and the χ^2^ test for dichotomous variables.

To determine the discriminative values of the RRI (preoperative, postoperative, and dRRI) and other potential predictors of CSA-AKI, receiver operating characteristics (ROC) curves were established. The area under the curve (AUC) was determined with the optimal cut-off for sensitivity and specificity using Youden’s J statistic. A backward stepwise multivariable regression analysis was computed to determine the independent predictors of AKI, including significant variables as confounders with a maximum of n/10 variables. A multivariate model was identified by applying a p-entry and removal of less than 0.05. Collinearity and interactions were tested and the Hoshmer–Lemeshow test was used to check the goodness-of-fit of the model. *p*-values < 0.05 were considered significant.

All statistical analyses were performed using MedCalc^®^ Statistical Software version 23.0.6 (MedCalc Software Ltd., Ostend, Belgium; https://www.medcalc.org; 28 October 2024).

## 3. Results

**Figure 2:**
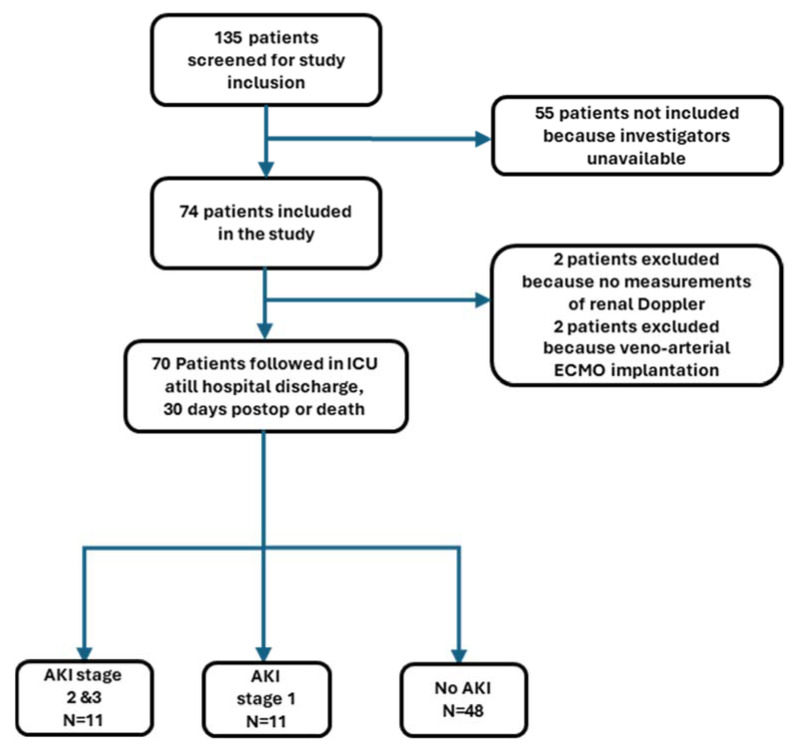
Flow chart of patient study selection. AKI, acute kidney injury; ECMO, extracorporeal membrane oxygenation; ICU, intensive care unit.

In this prospective study, 135 patients were screened, 74 were enrolled, 70 remained in the final analysis, and 4 patients were secondarily excluded (Figure 2).

Within the first five postoperative days, 22 patients developed CSA-AKI, of whom 11 developed stage 1 and 11 stage 2 or 3. The baseline characteristics and intraoperative data are displayed in Table 2 and Table 3. Compared with patients with no or mild CSA-AKI (AKI-0/1), those with moderate to severe CSA-AKI (AKI 2/3), were older, exhibited higher Cleveland score values, had lower hematocrit levels, and were more likely to receive a blood transfusion and inotropic support after weaning from CPB. The type of surgery, duration of aortic clamping, and intraoperative hemodynamic parameters did not differ between the two groups.

Postoperatively, LCOS developed in three and two patients with CSA-AKI-2/3 and CSA-AKI-0/1, respectively (27.3% vs. 3.4%, *p* = 0.045) The hospital length of stay was longer for patients with CSA-AKI-2/3 than those with CSA-AKI-0/1 (28 [15] days vs. 10 [2.4] days, *p* = 0.016). Five patients with CSA-AKI-2/3 died within 30 days after surgery whereas all patients with CSA-AKI-0/1 survived until discharge or 30 days after surgery. Among the surviving patients with AKI-2/3 at 30 days after surgery, two required renal replacement therapy.

**Figure 3:**
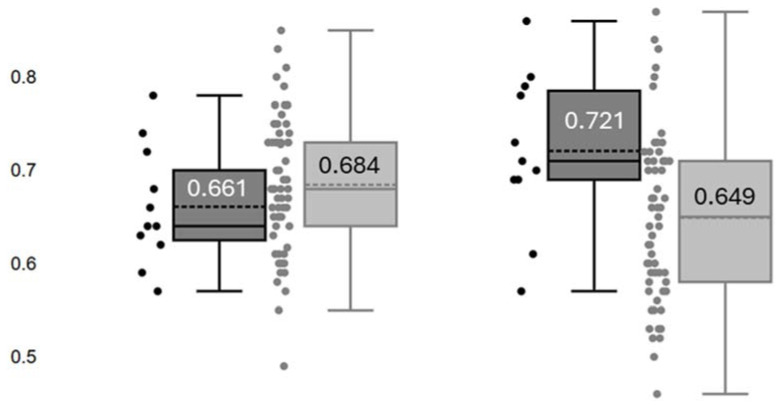
Renal resistance index (RRI) determined before surgery (**Left**) and at admission in the intensive care unit (**Right**) in patients with no AKI or stage 1 AKI (■) and those with stage 2 or 3 AKI (■). Numbers in each box refer to the mean values of RRI.

Preoperative and postoperative RRI are illustrated in Figure 3. Preoperative RRI did not differ between subjects with AKI-0/1 and those with AKI-2/3 (*p* = 0.290). Compared with the preoperative measurements, postoperative RRI increased by a mean of 10.9% in patients with AKI-2/3 (*p* = 0.004), while it decreased by a mean of −5.3% in those with AKI-0/1 (*p* < 0.001).

**Figure 4:**
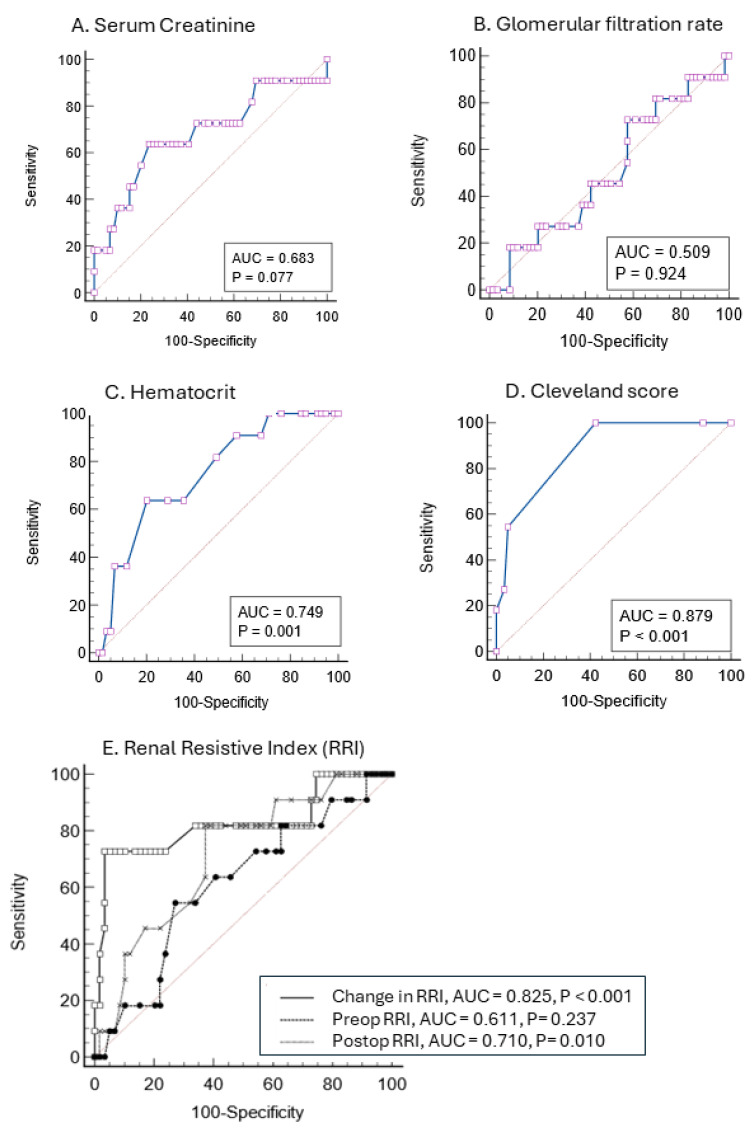
Prediction of postoperative stage 2 and 3 Acute kidney injury with receiver operating characteristic (ROC) curves based on serum creatinine (**A**), glomerular filtration rate (**B**), hematocrit (**C**), the Cleveland score (**D**), and calculation of preoperative, postoperative and changes in the renal resistance index (**E**). The area under the curve (AUC) appears in cartouche with *p* value.

The ROC-AUC of preoperative serum creatinine and both eGFR-CG and eGFR-MDRD showed poor discrimination for predicting CSA-AKI-2/3 (AUC 0.683, 0.509, and 0.610, respectively). As shown in Figure 4, hematocrit levels provided intermediate discrimination (AUC of 0.749) while the Cleveland score offered higher discrimination with a ROC-AUC of 0.879 (sensitivity 100% and specificity 57%). The ROC-AUC of postoperative RRI to predict CSA-AKI-2/3 was 0.710 (sensitivity 81.8% and specificity 62.7%) with an optimal cut-off point for discrimination at 0.68. The ROC of the dRRI provided better discrimination with an AUC of 0.825 (sensitivity 72.7% and specificity 96.6%) and an optimal cut-off point at 9.4% (positive and negative prediction values of 80% and 95%, respectively).

The multivariate regression analysis yielded three independent predictors for AKI-2/3, the Cleveland score, dRRI, and the need for combined inotropic support (Table 4). This model has a ROC-AUC of 0.917 (95% CI 0.825–0.970).

## 4. Discussion

In this prospective, unblinded, observational pilot study, our data confirm the usefulness of the Cleveland score to predict CSA-AKI (stage 2 and 3) while the changes in perioperative Doppler-derived RRI were helpful to identify the ongoing process of CSA-AKI.

### 4.1. Relationship with Previous Studies

In agreement with previous reports [1], we found a 31.4% incidence of CSA-AKI, with 50% being mild and transient (stage 1) and the other persisting till ICU discharge or death. The whole study population was considered at moderate risk of major perioperative complications owing to a mean EuroSCORE of 5.2 and a high prevalence of diabetes mellitus (44.3%) and heart failure (28.6%). The predominance of Afro-Caribbean ethnicity (79%) represented an additional risk factor for CSA-AKI as reported from a recent analysis of the US 2001–2020 National Inpatient Sample [15].

Various simple tools have been proposed to identify and monitor patients at risk for AKI, namely serum creatinine, and estimation of GFR using the Gault–Cockroft formula and the more recent MDRD equation [5]. Diagnosis of kidney dysfunction using these biomarkers usually lags by 24 to 48 h after the onset of renal insult [1]. In our study, plasma creatinine levels and both eGFR-CG and eGFR-MDRD failed to predict CSA-AKI (AUC < 0.7). In contrast, a composite tool such as the Cleveland score that incorporates preoperative clinical and biological variables has been validated to accurately predict the advanced severe stage of AKI that necessitates renal replacement therapy after open-heart surgery [10]. In the current study, the Cleveland score largely outperformed both preoperative serum creatinine and postoperative RRI whereas chronic treatments (i.e., inhibitors of the renin-angiotensin system) and creatinine clearance derived from the Cockroft–Gault equation were unrelated to the development of CSA-AKI-2/3. Interestingly, preoperative anemia and LCOS were also associated with the development of AKI-2/3. After multivariate regression analysis, only the Cleveland score, dRRI, and the need for dual inotropic support remained independent risk factors of CSA-AKI-2/3.

Determination of RRI upon admission in the ICU was a moderate marker of AKI-2/3 with an ROC-AUC of 0.71 and an optimal cut-off value at 0.68 (sensitivity of 81.8% and specificity of 62.7%). A meta-analysis of 18 trials including 1656 patients, supports the contention that early postoperative Doppler-derived RRI may help to identify AKI in critically ill patients admitted in the ICU, with better discrimination among patients undergoing surgical procedures than in those treated for sepsis (AUC of 0.88 vs. 0.80, respectively) [16]. The median diagnostic cut-off for RRI varied between 0.68 and 0.74 as a result of the population heterogeneity (e.g., preexisting chronic kidney disease, vascular impairment, sepsis, mixed surgical, and medical population) [8]. In line with these data, another meta-analysis focusing on surgical patients (10 trials, N = 911) indicated better performance of postoperative rather than preoperative RRI measurements to predict AKI, particularly for the persistent forms [17].

Although postoperative determination of RRIs convey more relevant information to identify CSA-AKI, large variations in ROC-AUCs (from 0.61 to 0.93), and cut-off values (from 0.68 to 0.85) are also related to different patient population, type of cardiac procedures as well as the prevalence and definition criteria of AKI (Table 5) [18,19,20,21,22,23,24,25].

Recently, preoperative determination of the renal functional reserve by calculating RRI after protein loading or abdominal compression has been shown effective in identifying patients prone to develop AKI after major surgery [26,27].

### 4.2. Study Implications

In this study, perioperative RRI measurements demonstrated opposite changes in the two subgroups: RRI increasing by a mean of 10.9% in CSA-AKI 2/3 and decreasing by a mean of 5.3% in CSA-AKI 0/1 patients. The clinical relevance of dRRI measured in each surgical patient resides in its high negative predictive value suggesting that the risk of CSA-AKI is very low when the dRRI is below 9.4%. The Doppler-derived RRIs integrate the combined effects of circulatory volume, intra-abdominal pressure, and arterial and venous pressure as well as extrinsic and intrinsic renal regulation [28]. Hence, the ability to maintain or decrease RRI postoperatively (CSA-AKI 0/1 group), reflects the physiological adaptive mechanisms involving myogenic autoregulation, tubulo-glomerular feedback, and endogenous vasodilators (i.e., nitric oxide, adenosine, eicosanoids) that tend to preserve renal blood flow and mitigate the neuroendocrine and inflammatory responses [7]. Conversely, the increased dRRI (>9.3%) in patients with CSA-AKI 2/3, reflects the inability of the kidneys to adapt to the stressful events owing to preexisting poor renal functional reserve and/or the high burden of vasoconstrictive agents and ischemic/inflammatory insults [8].

## 5. Study Strengths and Limitations

The aim of this observational study was to question the impact of perioperative determination of RRI in AKI risk assessment. Other strengths include the prospective design and assessment of multiple additional baseline variables.

Some limitations of this study should be acknowledged. First, this single-center study included a small sample of patients undergoing a variety of cardiac interventions. The lack of statistical power precluded the analysis of potential risk factors (i.e., duration of aortic clamping, treatment with inhibitors of the renin-angiotensin system, advanced age, and preexisting renal dysfunction) and to assess the added value of RRI. Secondly, we did not provide continuous hemodynamic data, although perioperative hypotension is a well-documented risk factor of CSA-AKI. Nevertheless, the higher incidence of LCOS in the CSA-AKI-2/3 group suggests unstable circulatory conditions among these patients that contributed to CSA-AKI. Thirdly, the Doppler-derived RRI determination is prone to operator-dependent error. Although intra- and interobserver variability was not determined, the two operators demonstrated a large experience with ultrasound measurements and previous studies indicated variability coefficients below 10% [19]. Finally, as we did not reassess the Doppler-derived RRI before hospital discharge, we were unable to a detect reversible or persistent changes in the RRI for patients recovering or not from CSA-AKI.

## 6. Conclusions

Perioperative determination of the changes in Doppler-derived RRI is helpful in detecting patients at risk of CSA-AKI. Our data indicate that postoperative increases in RRI are predictive of moderate-to-severe AKI.

Artificial intelligence, machine learning, and bioinformatics should be used to screen a large databases of cardiac surgical patients and update the prediction model for AKI risk stratification. Future trials should also evaluate hemodynamic and pharmacologic goal-directed interventions to reverse elevated RRI and prevent the ongoing process of AKI [29].

## Figures and Tables

**Table 1:** Definition criteria of the RIFLE score. AKI, acute kidney injury.

Stage		Serum Creatinine ↑ or GFR ↓	Urine Output ↓
RISK	AKI-1	150–200% or >25–50%	<0.5 mL/kg/min for 6 h
Injury	AKI-2	200–300% or >50–75%	<0.5 mL/kg/min for 12 h
Failure	AKI-3	>300% or >75% or >4 mg/dL	<0.3 mL/kg/min for 24 h or anuria > 12 h
Loss		Persistent AKI = Complete loss of renal function > 4 weeks	
ESRD		End-stage renal disease = Complete loss of renal function > 4 weeks	

**Table 2:** Baseline patient characteristics. Data are expressed as number (percentage) and mean (standard deviation). GFR-CG, glomerular filtration rate derived from Cockroft-Gault formula; MDRD, modification in diet of renal disease.

Characteristics	All Patients N = 70	AKI Stage II–III N = 11	AKI Stage 0–I N = 59	*p*-Value
Age, years	65 (11)	72 (7)	64 (12)	0.045
Woman	21 (30)	4 (36)	17 (29)	0.248
Body Mass Index, kg/m^2^	25.4 (3.5)	25.7 (3.5)	25.3 (3.5)	0.803
Caucasian/Afro-caribbean/Other	11/55/4	3/7/1	38/48/3	0.765
**Comorbidities**				
EuroSCORE II	5.2 (2.7)	6.3 (2.7)	4.7 (2.7)	0.188
Cleveland score	2.7 (0.9)	4.2(1.2)	2.4 (0.7)	0.003
Hypertension, n	45 (64.3)	9 (81.8)	36 (61.0)	0.189
Diabetes mellitus, n	31 (44.3)	6(54.5)	25 (42.4)	0.459
Insulin-dependent	12 (17.1)	2(18.2)	10 (16.9)	0.876
Non-insulin-dependent	19 (27.1)	4 (36.4)	15 (25.4)	0.343
Coronary artery disease	39 (55,7)	7 (63.6)	32 (54.2)	0.567
Atrial fibrillation	7 (10.0)	0 (0.0)	7 (11.9)	0.260
Heart Failure	20 (28.6)	4 (36.4)	16 (27.1)	0.536
Pulmonary hypertension, n	11 (15.7)	2 (18.2)	9 (15.3)	0.807
Peripheral arterial disease, n	14 (20.0)	3 (27.3)	11 (18.6)	0.514
Current smoking	14 (20.0)	2 (18.2)	12 (20.3)	0.870
Chronic obstructive pulmonary disease	5 (7.1)	1 (9.1)	4 (6.8)	0.811
Chronic alcohol consumption	16 (22.9)	2 (18.2)	14 (23.7)	0.689
**Medications**				
Beta-blockers	45 (64.3)	7 (63.6)	38 (64.4)	0.986
Angiotensin Converting Enzyme Inhibitors or Angiotensin II Antagonists	43 (61.4)	7 (63.6)	36 (61.0)	0.9821
Diuretics	21 (30)	4 (36.4)	17 (28.8)	0.618
Statines	39 (55.7)	7 (63.6)	32 (54.2)	0.786
**Laboratory**				
Creatinine, mcg/ml	95.5 (20.8)	84.2 (124)	97.6 (19.7)	0.226
GFR-CG < 60 mL/min	27 (38.6)	4 (36.4)	23 (39.0)	0.823
GFR-MDRD < 60 mL/min/1.73 m^2^	19 (27.1)	3 (27.3)	16 (27.1)	0.968
Hematocrit,%	38.3 (3.9)	34.7 (3.3)	38.9 (3.7)	0.008

**Table 3:** Surgical and perioperative data. Variables are presented as the mean (SD), median (interquartile range), or number (percentage) as appropriate. CPB, cardiopulmonary bypass; IABCP, intraaortic balloon counterpulsation; LCOS, low cardiac output syndrome.

Characteristics	All N = 70	AKI Stage II–III N = 11	AKI Stage 0–I N = 59	*p*-Value
**Surgical characteristics**				
Emergency, n	9 (12.9)	2 (18.2)	7 (18.2)	0.986
Re-operation, n	3 (4.3)	1 (9.1)	2 (3.4)	0.395
Endocarditis, n	3 (4.3)	1 (9.1)	2 (3.4)	0.395
Coronary artery bypass surgery	32 (45.5)	5 (45.5)	27 (45.8)	0.985
Valvular replacement/repair, n	34 (48.6)	6 (54.5)	28 (47.5)	0.876
Combined surgery, n	4 (5.7)	0 (0.0)	4 (6.8)	0.377
Ascending aorta, n	3 (4.3)	1 (9.1)	2 (3.4)	0.395
Aortic clamping time, min	94 (34)	110 (31)	91 (34)	0.237
CPB time, min	142 (39)	159 (44)	139 (40)	0.290
**Hemodynamics**				
Mean Arterial pressure, mmHg				
Before CPB End of surgery	97 (16) 80 (12)	90 (14) 83 (11)	99 (16) 80 (13)	0.154 0.509
Heart rate, beat/min				
Before CPB End of surgery	76 (16) 88 (15)	83 (13) 94 (13)	74 (17) 86 (15)	0.109 0.188
Central Venous Pressure, mmHg				
Before CPB End of surgery	7.8 (2.5) 10.6 (2.6)	7.6 (2.9) 12.3 (1.3)	7.8 (2.5) 10.3 (2.8)	0.892 0.019
Arterial lactate, mmole/L				
End of surgery	2.8 (1.0)	3.6 (1,7)	2.7 (0.8)	0.183
**Circulatory support after CPB**				
IABCP, n	4 (5.7)	2 (18.2)	2 (3.4)	0.054
Dobutamine, n	24 (34.3)	7 (63.6)	17 (28.8)	0.027
Adrenaline, n	5 (7,1)	3 (27.3)	2 (3.4)	0.005
Noradrenaline, n	24 (34.3)	5 (45.5)	19 (32.2)	0.398
LCOS, n	5 (7.1)	3 (27.3)	2 (3.4)	0.045
**Red blood cell transfusion**				
Patient, n	11 (15.7)	5 (45.5)	6 (10.2)	0.003
Red blood cell, unit	0 (0–4)	1 (0–5)	0 (0–4)	0.111

**Table 4:** Multivariate stepwise regression analysis for stage 2 and 3 acute kidney injury. IABCP, intraaortic balloon counterpulsation.

	Odds Ratio	95% CI	*p*-Value
Cleveland Score	2.93	1.14–7.50	0.025
Change in Renal Resistive Index	1.13	1.02–1.25	0.016
Need> or =2 inotropes or IABCP	22.8	1.19–434.1	0.037
Variables included	Cleveland score, dRRI, need ≥2 inotropes/ABCP, hematocrit, age, creatinine clearance, transfusion		
Variables removed	hematocrit, age, creatinine clearance, transfusion		
N	70		
Hosmer Lemeshow	6.819 (df = 3, *p* = 0.556)		
Nagelkerke R^2^	0.616		

**Table 5:** Characteristics of studies testing the performance of the renal resistive index (RRI) to predict acute kidney injury after cardiac surgery. AKIN, acute kidney injury network; CABGS, coronary artery bypass graft surgery; dRRI, percentage change from preoperative to postoperative measure of renal resistive index; RIFLE, risk, injury, failure, loss of kidney function, and end-stage kidney failure; TAAD, acute type A aortic dissection; TAVI, transcatheter aortic valve implantation; VR/Vr, valve replacement/valve repair.

Study Year	N	Surgery	Criteria AKI	Prevalence AKI, %	Mean Age	RRI Measurement	Cut-Off	AUC	Se, %	Sp, %
Peillex 2020 [21]	100	TAVI	AKIN Cystatin	10 10	84	Preoperative Postop day 1 Postop day 3	NA 0.79 NA	NA 0.766 NA	NA 80 NA	NA 62 NA
Sinning 2014 [24]	132	TAVI	AKIN	24.2	80	Preoperative Postop 4 h Postop day 1–7	NA 0.85 NA	NA 0.73 NA	NA 58 NA	NA 86 NA
Wu 2017 [25]	62	acute TAAD	AKIN	65	47	Preoperative Postop 6 h Postop day 1–3	NA 0.72 NA	NA 0.918 NA	NA 95 NA	NA 72 NA
Regolisti 2017 [23]	60	CABG VR/Vr	AKIN	38	69	Before incision End of surgery Postop 4 h–day 1	NA 0.67 NA	NA 0.710 NA	NA 67 NA	NA 64 NA
Guinot 2013 [19]	82	CABG VR/Vr	RIFLE	26	72	Preoperative Postop 0–2 h Postop 6 h Postop day 1	NA 0.73 NA NA	0.630 0.930 0.870 0.840	NA 93 NA NA	NA 88 NA NA
Bossard 2011 [18]	65	CABG VR/Vr	>30% sCreat	28	70	Postop 0–2 h	0.74	0.910	89	91
Hertzberg 2017 [20]	96	Mixed	AKIN	28	69	Preoperative	0.70	NA	78	46
Quin 2017 [22]	67	acute TAAD	AKIN	31	46	Postop 6 h	0.72	0.855	91	71
Current study 2024	70	CABG VR/Vr	RIFLE	16 (AKI-2/3)	65	Preoperative Postop 0–2 h dRRI pre-post	0.64 0.68 9.4	0.610 0.710 0.830	55 82 73	73 63 97

## Data Availability

The data presented in this study are available on request from the corresponding author. The data are not publicly available due to privacy.
